# Effects of CMV on T and NK Cells; With Emphasis on Immunometabolism

**DOI:** 10.1155/bmri/7838997

**Published:** 2026-04-13

**Authors:** Haideh Namdari, Mohsen keshavarz, Maryam Hosseini, Farzad Parvizpour, Asrin Emami, Elahe Izadi, Milad Shahbazi Asl, Mehdi Shahgolzari, Farhad Rezaei

**Affiliations:** ^1^ Iranian Tissue Bank and Research Center, Gene, Cell and Tissue Research Institute, Tehran University of Medical sciences, Tehran, Iran, tums.ac.ir; ^2^ The Persian Gulf Tropical Medicine Research Center, The Persian Gulf Biomedical Sciences Research Institute, Bushehr University of Medical Sciences, Bushehr, Iran, bpums.ac.ir; ^3^ Trauma Research Center, Shahid Rajaee (Emtiaz) Trauma Hospital, Shiraz University of Medical Sciences, Shiraz, Iran, sums.ac.ir; ^4^ Department of Molecular Medicine, Faculty of Medicine, Kurdistan University of Medical Sciences, Sanandaj, Iran, muk.ac.ir; ^5^ Department of Microbiology and Immunology, Faculty of Veterinary Medicine, University of Tehran, Tehran, Iran, ut.ac.ir; ^6^ Department of Medical Nanotechnology, School of Medicine, Zanjan University of Medical Sciences, Zanjan, Iran, zums.ac.ir; ^7^ Virology Department, School of Public Health, Tehran University of Medical Sciences, Tehran, Iran, tums.ac.ir

**Keywords:** ATP, CMV, glycolysis, immunometabolism, phosphorylation

## Abstract

CMV is masterful at manipulating stress responses and conducting them in a way that yields beneficial outcomes. CMV always causes metabolic changes in the infected cells. However, nutritional status affects the pattern of events following CMV infection. Oxidative phosphorylation (OXPHOS) and glycolysis are two vital pathways for energy generation in mammalian cells, which differ in terms of ATP production and location. Understanding metabolic alterations required for CMV infection may lead to the design of novel therapeutic methods based on targeted inhibition of these cellular metabolic pathways. This review explores how CMV mimics, exploits, or interferes with the host cell, with emphasis on immunometabolism, and how, in doing so, it may evade immune responses.

## 1. Introduction

During an era, in which a plethora of advances in transplantation have transpired, an unresolved issue remains incessant: human cytomegalovirus (CMV) infections [1]. Albeit 60%–100% of adults are infected by the virus, they remain asymptomatic [2]. The inaugural infection with CMV, as an opportunist virus, is oftentimes mild and manifests no symptoms, which enable it to evade the host’s immune response; however, it can beget a persistent or latent infection [3]. The full pathogenicity of CMV only comes to light when immunocompromised individuals are involved [4]. Both arms of the immune system, innate and adaptive, are enmeshed in CMV infection as CD8+, CD4+, *γδ* T‐cells, and Natural killer (NK) cells play a significant role in this situation [5–7]. It is clear that CMV drastically alters the metabolic status of the host cell, which is required for successful infection [8]. The success rate of an anti‐CMV response is conspicuously imbued in cellular metabolism [9]. Metabolism is the biochemical process used by cells to produce essential biomolecules and energy production (i.e., Adenosine Triphosphate [ATP]). Two main pathways are used for generating ATP from metabolic fuels including glycolysis and mitochondrial oxidative phosphorylation (OXPHOS) [10, 11]. Glucose fuels both pathways, however; glutamine and fatty acids (FAs) can also be considered as other initial substances for OXPHOS [12]. In the context of immunity, proper metabolic changes are necessary in immune cells to guarantee an effective immune response. Glycolysis through the conversion of glucose to pyruvate through metabolic reaction sequences is a dominant energy supply pathway in pro‐inflammatory cells [13]. This process is oxygen‐independent and favored by pro‐inflammatory cells due to its rapid activation through the induction of glycolytic enzymes, which mediates cell biosynthesis [14]. The innovatory leap in technologies has provided unprecedented insight into immune cells and their top‐tier “bioenergy management”. This review intends to explore how chief effectors in anti‐CMV immunological response, T and NK cells, are impacted, and in what ways their metabolic pathways are altered during CMV infection.

## 2. Importance of the Immune System in CMV Infection

The immune system plays a critical role in controlling CMV replication, preventing reactivation, and minimizing disease progression by activating T‐cells and NK cells [15]. NK cells, as the first line of defense, are identified as crucial for suppressing human CMV through their rapid production of IFN‐*γ*, granzymes, and perforins, as well as performing antibody‐dependent cellular cytotoxicity (ADCC) against CMV infection [16, 17]. However, the ability of CMV to evade the innate immune system highlights the crucial role of adaptive immunity in achieving long‐term viral control. NK cells are a link in innate‐adaptive cell‐mediated immunity by stimulating the CD8+ cytotoxic T lymphocytes (CTLs) response through secretion of IFN‐*γ* [18].

CTLs are essential for identifying and destroying CMV‐infected cells, particularly during reactivation events. CD4+ helper T‐cells provide vital support by promoting the activation and maintenance of CTLs and by aiding B‐cells in producing neutralizing antibodies [18–21]. In immunocompromised individuals, such as organ transplant recipients or those with HIV, weakened T‐cell responses often lead to unchecked CMV replication and severe complications [19–21].

## 3. NK Cell Immunometabolism During Rest and Following Activation

**Figure 1:**
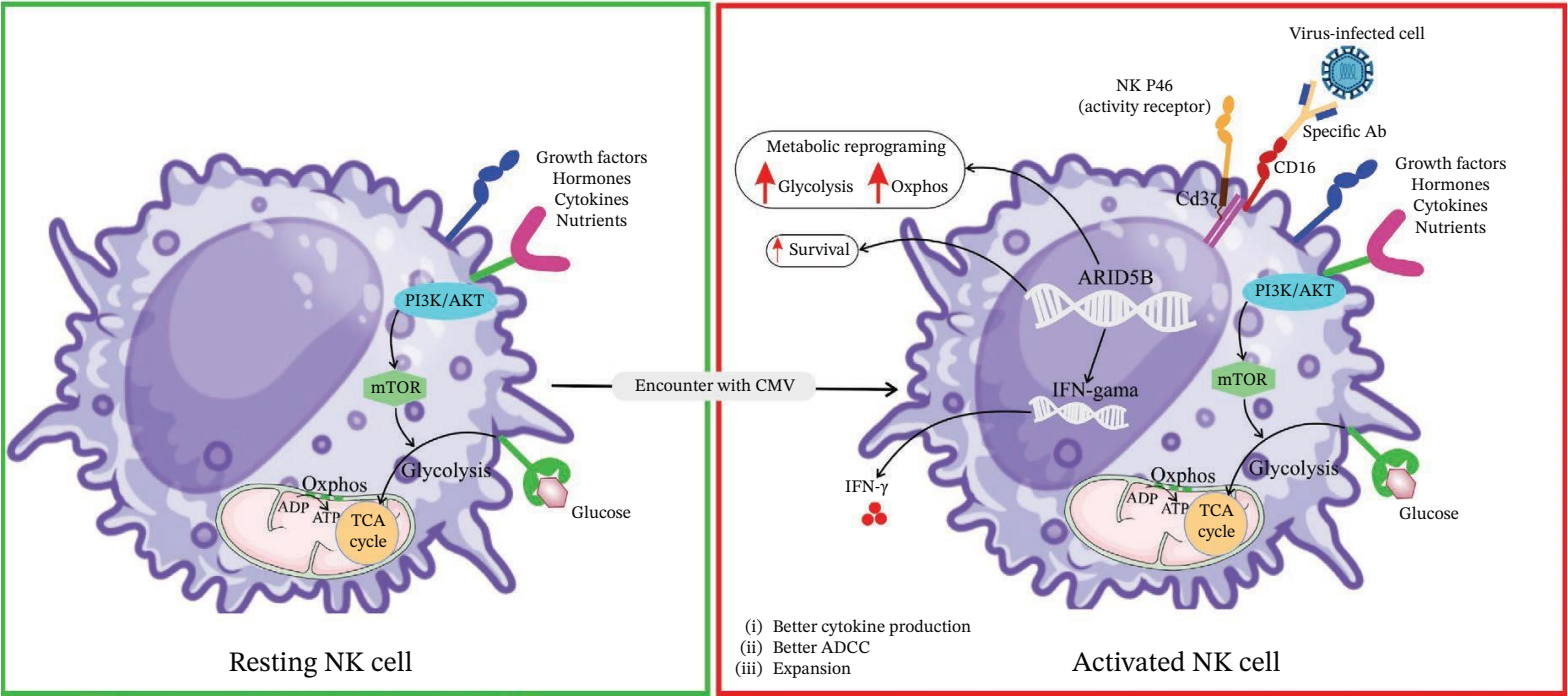
Molecular factors governing adaptive NK cell function. The Fc segment of antibodies (Abs) signals adaptive NK cells to recognize target cells through binding to CD16. NKp46 triggering mediates signaling through its association with immunoreceptor tyrosine‐based activation motif (ITAM)‐bearing molecules CD3*ζ* and CD16, leading to NK cell activation. Epigenetic programming, following the activation of adaptive NK cells, promotes upregulation of ARID5B in the promoter region, driving abundant IFN‐*γ* secretion, altering metabolic reprogramming, and prolonging survival.

Changes in metabolism are crucial for fueling the response of NK cells, and altered metabolism is associated with the dysfunction of NK cells [22]. Mainly, mature resting NK cells utilize the OXPHOS pathway to meet their homeostatic requirements. This approach makes sense because it allows them to produce energy efficiently without investing excessive energy in the synthesis of biochemical molecules [23]. Following the activation of NK cells, metabolism is subjected to change. In this regard, a study by Keating et al. has demonstrated that NK cells strongly upregulate both glycolysis and the OXPHOS pathways in responses to a variety of cytokines (such as Interleukin‐2 [IL‐2] or a combination of IL‐12/15), leading to better functionality, like cell cytotoxicity and Interferon (IFN)‐*γ* production [24] (Figure 1). It has been suggested that for IFN‐*γ* production, one important step that is triggered by glucose is the unbinding of glyceraldehyde‐3‐phosphate dehydrogenase (GAPDH) from the 3′ UTR of IFN‐*γ* mRNA [25].

The most consistent finding thus far is that the mammalian target of rapamycin (mTOR), a serine‐threonine kinase, is a key metabolism regulator in NK cells, and rapamycin, a potent inhibitor of mTORC1, efficiently inhibits glycolysis in NK cells [26]. Human NK cells are broadly classified into two main subsets: (a) CD56^dim^ and (b) CD56^bright^ cells, which differ significantly in their metabolic profiles. The CD56^bright^ subset primarily arises from peripheral blood and secondary lymphoid tissues and is characterized by potent cytokine production and heightened metabolic responsiveness [27]. This enhanced metabolic activity in CD56^bright^ cells is likely driven by their elevated expression of cytokine receptors, enabling them to regulate various metabolic pathways more robustly than CD56^dim^ cells following cytokine stimulation [24].

Accordingly, it has been shown that CD56^bright^ NK cells have higher mTOR activity and exhibit stronger metabolic alterations in responses to IL‐2 or IL‐12/IL‐15 stimulation in comparison with CD56^dim^ NK cells [12]. Furthermore, direct glycolysis inhibition of murine NK cells, by 2‐Deoxyglucose (DG) or galactose, prevented granzyme B and IFN‐*γ* expression levels after stimulation through Toll‐like receptor‐, cytokine‐, or stimulatory receptors [28]. This further emphasizes the importance of the glycolysis pathway in activated NK cells. In addition to the above‐mentioned pathways, data show that upon activation of NK cells nutrient uptake, iron, and amino acids, will be increased [22]. Hence, cytokine stimulation of NK cells leads to an augmentation of amino acid transporter SLC7a5 and a subsequent increase of glutamine uptake. Of note, SLC7a5 is key for the activation and maintenance of c‐Myc signaling, which contributes to the regulation of the glycolytic pathway of NK cells [26].

## 4. T Cell Immunometabolism During Rest and Following Activation

During the resting period, T cells require only basal replacement biosynthesis to fuel the OXPHOS pathway and rely on basic levels of glucose and FA metabolism [29]. To change between different activation statuses, naïve, effector, and memory, T cells require active reprogramming of cellular metabolism [30]. One important observation after primary stimulation of T cells, through cytokines like IL‐2, CD28 ligation, and the Pl3K‐dependent activation of the Akt pathway, is an increase in cell size along with a metabolic shift to glycolysis, which provides the necessary energy for effector functions [31, 32]. Activated Akt can potentiate the mTOR path, a key regulator of translation [33], and also stimulates glycolysis by increasing the activity of glycolytic enzymes and upregulating the expression of nutrient transporters [31]. Noteworthy, lactate dehydrogenase A (LDHA) in T cells maintains a high concentration of acetyl‐coenzyme A to enhance histone acetylation and the transcription of IFN*γ* [34]. Ablation of LDHA in T cells gives rise to the prevention of IFN‐*γ* production after activation through augmentation of OXPHOS and a subsequent drop in acetyl‐Co A‐mediated histone acetylation at the enhancer and promoter of the IFN*γ* gene [34].

## 5. How CMV Alters Metabolism of the Following Cells

### 5.1. NK Cells

Following CMV infection, NK cells undergo durable phenotypic reprogramming driven by epigenetic remodeling, giving rise to adaptive NK cells. New studies have confirmed that canonical (CMV naïve) compared with adaptive (CMV experienced) NK cells show different metabolisms and targeting these pathways may be considered a therapeutic value [35]. These cells are predominantly CD56^dim^CD57^+^NKG2C^+^, typically lacking signaling adaptors such as Fc*ε*R*γ*, Syk, PLZF, and also often losing the inhibitory receptor NKR‐P1A (CD161) [36, 37]. This stable signature reflects chromatin‐level changes, including DNA hypomethylation, which supports their long‐term persistence and enhanced functional capacity [38]. These adaptive NK cells clonally expand and can persist for years post‐infection, appearing not only in peripheral blood but also redistributed to tissues such as the liver, lung, spleen, and even tumor environments. Recent single‐cell profiling studies have identified transitional NK subsets (e.g., CD56^bright^ NKG2C^+^NKG2A^+^ (A^+^C^+^) NK cells) that may act as precursors to fully adaptive states, suggesting a developmental gradient shaped by CMV [39]. Functionally, CMV‐adaptive NK cells exhibit potent CD16‐mediated ADCC, driven by mTOR‐dependent metabolic reprogramming, and produce robust IFN‐*γ* responses upon antibody‐dependent stimulation [38]. They show reduced sensitivity to cytokines like IL‐12/IL‐18, consistent with their silencing of associated signaling pathways [38]. Metabolically, adaptive NK cells demonstrate elevated glycolysis and OXPHOS, underpinned by chromatin remodelers such as ARID5B, which promotes mitochondrial biogenesis and increased respiratory capacity [35]. Given that IL‐15 can shift NK cell metabolism toward glycolysis, using IL‐15 superagonist, ALT‐803, has shown promising results in the treatment of recurrent CMV reactivation after hematopoietic cell transplant [34]. They also revealed that in addition to higher glycolytic capacity, CD56^dim^CD57^+^NKG2C^+^ CMV‐adaptive NK cells can increase the expression of genes involved in lipid catabolism to meet satisfied energy [35]. These altered metabolic states support their longevity and rapid recall responses. Collectively, the combined insights from recent studies reveal a highly specialized NK cell subset that is epigenetically imprinted by CMV, redistributed to key tissues, metabolically primed, and functionally optimized for antibody‐dependent and antiviral responses. These advancements underscore the therapeutic potential of harnessing adaptive NK cells in antiviral and oncologic immunotherapies. Importantly, the metabolic and functional characteristics observed in adaptive NK cells should not be interpreted as direct consequences of acute CMV infection. Rather, they arise predominantly in CMV‐seropositive individuals as a result of long‐term clonal expansion and epigenetic reprogramming [40, 41]. These adaptations reflect a chronic “imprint” of CMV exposure rather than ongoing viral replication. The enhanced bioenergetic profile of adaptive NK cells—marked by increased glycolysis and mitochondrial respiration—is thus more accurately described as a consequence of persistent expansion and functional calibration within the CMV‐seropositive host [35, 42], rather than an immediate, transient outcome of infection. This distinction is essential for interpreting the biology of NK cell memory and its relevance to immune surveillance and immunotherapy.

### 5.2. T Cells

Following CMV infection, T cells undergo dynamic and long‐lasting changes in both function and metabolism. The early immune response is marked by the activation of CMV‐specific CD4^+^ T cells, which rapidly expand and secrete Th1‐type cytokines such as TNF‐*α* and IFN‐*γ* [43, 44]. This is followed by the activation and proliferation of highly cytotoxic CD8^+^ T cells, which can be detected in peripheral blood for days post‐infection and are essential for controlling viral replication and preventing reactivation. These CD8^+^ T cells maintain an effective cytotoxic phenotype even during the latent phase of CMV [45–47]. During the acute phase of infection, effector CD8^+^ T cells undergo a profound metabolic shift toward aerobic glycolysis (the Warburg effect) to meet the high biosynthetic and energetic demands of cytokine production, proliferation, and cytotoxic function [25, 48]. Glycolysis supports rapid ATP generation and facilitates effector functions such as IFN‐*γ* production. However, when glucose is limited, activated T cells demonstrate metabolic flexibility by transiently upregulating OXPHOS, highlighting their capacity to adapt to fluctuating nutrient availability [25]. These findings underscore the importance of both glycolytic and mitochondrial pathways in sustaining T cell responses during acute CMV infection. There is growing interest in the possibility of using metabolic modulators to enhance T cell energy availability during infection—preclinical models have shown that combining glycolysis‐promoting agents with mitochondrial boosters can improve lymphocyte function and viral control [49]. In contrast, CMV seropositivity is associated with long‐term adaptations in the T cell compartment, driven by chronic antigenic stimulation rather than active infection. One hallmark of CMV‐seropositive individuals is the accumulation of highly differentiated effector and memory T cell subsets, such as CD8^+^CD57^+^ and the terminally differentiated EMRA (effector memory RA) subset defined as CD8^+^CD45RA^+^CD27^-^[50–53] Metabolically, these long‐lived cells exhibit increased mitochondrial mass and elevated respiratory capacity, alongside a persistent glycolytic bias that sustains their cytotoxicity and survival [54–56]. Notably, many of these CMV‐driven T cells exhibit a senescence‐associated secretory phenotype (SASP), characterized by the secretion of inflammatory cytokines (e.g., IL‐6, IL‐1*β*) and tissue‐degrading enzymes. This SASP is tightly regulated by the p38 MAPK signaling pathway, which also suppresses autophagy via mTORC1‐independent mechanisms [57–59]. Despite signs of mitochondrial impairment in these senescent‐like T cells, they maintain high glycolytic flux to compensate for energetic needs, reflecting a shift toward glycolysis‐dependent survival. This bioenergetic remodeling is driven by long‐term transcriptional and epigenetic reprogramming, distinguishing it from the transient metabolic activation seen during acute infection [60, 61]. Together, these findings emphasize the importance of clearly distinguishing between the immediate metabolic reprogramming that supports effector T cell functions during acute CMV infection, and the long‐term bioenergetic adaptations seen in chronically stimulated T cells in CMV‐seropositive individuals. While the former is reversible and inflammation‐driven, the latter reflects durable functional imprinting shaped by persistent antigen exposure, with broad implications for immune aging, inflammation, and T cell‐based immunotherapies.

Despite the substantial roles of *γδ* T‐cells, especially V*δ*2^−^ *γ* T and V*γ*4V*δ*5 T‐ cells, against CMV‐infected cells, it seems there is no specialized investigation on metabolic consequences during activation of these types of cells in CMV infection responses.

## 6. How CMV Alters Immunometabolism Pathways to Manipulate Cytotoxic Effect of NK Cells and CD8^+^ T Cells

Cellular cytotoxicity is very major in immune responses. Both NK cells and T cells, particularly CMV‐specific CD8^+^ T cells, have a major role in the clearance of CMV infection through the killing of virus‐infected cells [48]. To kill virus‐infected cells, upon antigen recognition by cognate receptors on NK and T cells [48], intracellular signaling gives rise to granzyme and perforin release and apoptosis of CMV‐infected cells [62–64]. Most importantly, granzyme causes to cleavage of viral immediate‐early (IE) proteins IE1 and IE2 in CMV‐infected cells. Of note, the expression of early and late viral proteins necessary for the production of CMV infectious virus requires a coordinated cascade of transcriptional events that are triggered by these proteins [65].

Indeed, appropriate metabolic conversions in CD8^+^ T cells and NK cells are required to ensure a productive cytotoxic response [12, 66]. In NK cells, upregulation of glycolysis and OXPHOS is important for cytotoxicity [67]. It has been shown that granzyme B expression in NK cells can be reduced when glycolysis or citrate‐malate shuttle CMS is suppressed [28].

It has been recently shown that CMV may impair the cytotoxic function of CD8^+^ T cells by changing metabolic pathways. CMV expresses the UL36 gene that produces the vICA (viral inhibitor of caspase‐8 activation) protein [68, 69]. This protein inhibits Casp8‐induced apoptosis that prevents cytotoxicity of CD8^+^ T cells in infected cells [70].

In sum, the current knowledge about metabolic regulation of cellular cytotoxic function during CMV infection is still limited and more research is required to fully understand the impact of immunometabolism changes on releasing cytotoxic granules during CMV infection.

## 7. How CMV Alters Immunometabolism Pathways to Manipulate Co‐Stimulatory and Inhibitory Signals of NK and T Cells

T cell activation, expansion, differentiation, and finally its fate depend on co‐signaling receptors that positively (Co‐stimulator) or negatively (Co‐inhibitor) regulate T cells [71]. Co‐stimulatory signals, downstream of co‐stimulatory receptors CD28, CD137, OX40, CD40L, and CD27 (CD70), promote T cell effector function [72–76]. The main function of co‐stimulatory receptors is amplifying TCR signaling that impacts metabolic reprogramming. For instance, cell cycle progression and cytokine production are among the functions that co‐stimulatory receptors can amplify through MAPK and PI3K/Akt pathways [77].

**Figure 2:**
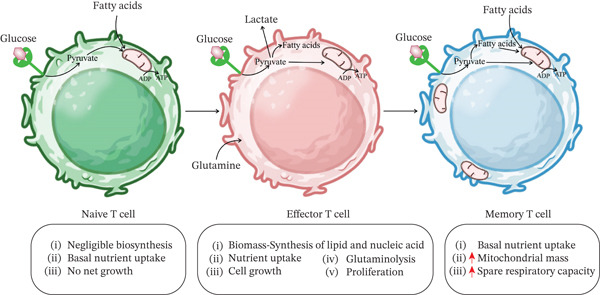
Different T cell subsets and their main metabolic characteristics. Naïve T cells primarily oxidize glucose and fatty acids in the mitochondria to generate ATP. During the transition from naïve to effector T cells, there is a significant increase in glucose uptake, with most of the glucose‐derived pyruvate being converted to lactate. Glutamine uptake also rises, and fatty acid synthesis is activated to support cellular activation. In memory T cells, mitochondrial mass increases, and a futile cycle of fatty acid synthesis and oxidation has been observed.

Following TCR and CD28 engagement with an antigen, glucose uptake increases to supply enough energy to maintain cell activation at the desirable rate by overexpression of glucose transporters, especially Glut1 [78, 79] (Figure 2). Recently, it has been shown that CMV decreases Glut1 and increases Glut4, 24 h after infection, which has threefold higher glucose transport capacity than Glut1 and results in more viral production [80]. Also, it has been demonstrated that CMV downregulates CD28 to dominate T‐cell activation and it may be the reason for glucose transporter replacement in infected cells [81].

CD58 is an adhesion molecule that has a crucial role in the co‐stimulation of NK and T cells to recognize CMV‐infected cells in collaboration with CD2 [82]. UL148 expressed by CMV interacts with CD58 and causes intracellular retention of CD58, impairing the ability of NK and T cells to be activated and fight against CMV [83]. However, their mechanism of action on metabolic pathways has not been identified yet.

Co‐inhibitory receptors, CTLA4 and PD‐1, oppose functional effects induced by TCRs and co‐stimulatory receptors and limit immune cell functions [84–86]. A growing number of studies have also shown that following CMV infection or reactivation, PD‐1 expression increases on the surface of NK cells, CD4^+^ and CD8^+^ T cells [87, 88]. Ligation of PD‐1 in T effector cells might reprogram them from aerobic glycolysis to catabolic lipid metabolism or fatty acid synthesis (FAS). This metabolic reprogramming can switch effector cells to Treg and T memory cells [89]. FAS increment is an obligatory stage in CMV infection because it is necessary for envelop synthesis [90]. Further, CTLA‐4 is overexpressed on T cells upon CMV infection inhibits cell cycle progression, and suppresses IL‐2 production [91]. Noteworthy, IL‐2 acts as an autocrine factor, activates, and amplifies T cell metabolic reprogramming toward a glycolytic phenotype. Following IL‐2 inhibition, in turn, metabolic reprogramming stops T‐cell proliferation and differentiation [92].

## 8. How CMV Alters Immunometabolism Pathways to Manipulate Chemotaxis (Migration) of NK and T Cells

In CMV infection, CD8^+^ and CD4^+^ T cells express specific types of chemokines and several proteins that show a critical role in chemotaxis and migration of cells [93, 94]. Alteration in chemokine receptor usage of CMV‐specific T cells might be required to constitute a protective immune response against CMV infection [95]. Accordingly, during primary infection, CCR7 expression on CMV‐specific T cells is downregulated, CCR1, CCR5, CXCR6, and CX3CR1 levels, by contrast, are highly expressed at the peak response [95, 96]. Of note, CX3CR1 is the only chemokine receptor expressed during latency. This receptor, through binding to fractalkine on endothelial cells of vessels, contributes to CMV latency [95]. Notably, during latency CMV‐specific CD8^+^ T cells do not express the chemokine receptor CCR7 [97]. Indeed, CMV‐specific CD4^+^ and CD8^+^ T cells show high‐level expression of CXCR3, which binds to CXCL10 on endothelial cells of vessels [95]. Therefore, effector CD8^+^ T cells are capable of migrating toward CXCL10 and play important roles in controlling CMV infection [98]. Concerning the NK cells, it should be noted that CMV has developed various strategies to evade the activity of these cells, among them, viral mimicry of chemokines [99, 100]. CMV encodes viral CXC‐ligand 1 (vCXCL1; UL146 gene product), which is expressed in the virus life cycle and is released from the infected cells. vCXCL1 binds to CXCR1 and CX3CR1 on NK cells and mediates the migratory pattern of CD56^dim^ cells to the site of CMV infection [101]. However, CMV‐infected cells can counterattack the NK cells due to the various immune evasion mechanisms they have developed [101]. Interestingly, it has been demonstrated that CMV infection overshadows the migratory behavior of NK cells in various contexts. In accordance, one recent study showed that CX3CR1^+^/CXCR1^+^ NK cells preferably migrate from inflamed tissues to the peripheral blood in response to acute exercise in CMV‐positive participants [80]. Further, one more recent in vitro study has suggested that during HCMV reactivation, adaptive NK cells by increased secretion of chemokines including CXCL10/IP‐10 and CCL4, play an important role in the recruitment of other immune cells such as T cells [102]. To date, there have been no studies that reveal a possible cellular metabolic basis for the altered chemotaxis of T and NK cells during CMV infection, and is worthy of further study.

## 9. CMV‐Induced Metabolic Alterations and Disease Susceptibility

Metabolic changes in immune cells caused by CMV infection are crucial in influencing physiology, immune responses, and disease susceptibility during the infection. Prolonged metabolic stress induced by CMV can result in functional exhaustion of NK cells and CTLs, diminishing their cytotoxic efficiency and compromising immune surveillance [15, 103].

Additionally, the accumulation of metabolic byproducts and chronic immune activation can lead to tissue damage or fibrosis, further affecting organ physiology [104].

CMV infection can lead to metabolic alterations in NK cells and CTLs that contribute to low‐grade chronic inflammation, which increases susceptibility to cardiovascular diseases, diabetes, and other metabolic disorders [105–107].

Additionally, CMV drives immune aging (immunosenescence) by inducing the persistent activation and clonal expansion of specific NK and CTL subsets, which reduces the diversity and responsiveness of the immune repertoire [108]. This immune dysfunction results in a higher susceptibility to opportunistic infections due to the inability of metabolically exhausted immune cells to effectively combat secondary pathogens [109]. Furthermore, impaired cytotoxicity in NK cells and CTLs, stemming from metabolic dysfunction, may enable tumor cells to escape immune surveillance, thereby elevating the risk of cancer development [110–112].

## 10. Conclusion

CMV infection can interfere with immune responses. Since CMV infection/reactivation in immunocompromised individuals is a global concern, research on designing novel therapeutic modalities is of particular importance. Based on recent studies, CMV is capable of manipulating various metabolic pathways in immune cells. This provides new insight that host cell metabolism can be used as a therapeutic target to inhibit CMV infection/reactivation. Thus, a clear understanding of the metabolic alterations during CMV infection would be tremendously helpful for therapeutic purposes.

## Nomenclature

ARID5B: AT‐rich interaction domain 5B
ATP: adenosine triphosphate
CMV: cytomegalovirus
FAs: fatty acids
IFN: interferon
LDHA: lactate dehydrogenase A
mTOR: mammalian target of rapamycin
NK: natural killer
OXPHOS: oxidative phosphorylation

## Funding

No funding was received for this manuscript.

## Conflicts of Interest

The authors declare no conflicts of interest.

## Data Availability

All data extracted and reported are included in this published article.
